# 

**Published:** 1891-01-10

**Authors:** 


PRICE ONE PENNY.

No. 224, Vol. IX.—January 10th, 1891.

[Registered at the General Post Office as a Newspaper for transmission in the United Kingdom and Abroad.]

WITH EXTRA NURSING SUPPLEMENT

Per Annum, 6s. 6d.; post free.

Monthly Parts, 6d.; post free 8d.

Ditto per Annum, 8s., post free.

### THE HOSPITAL

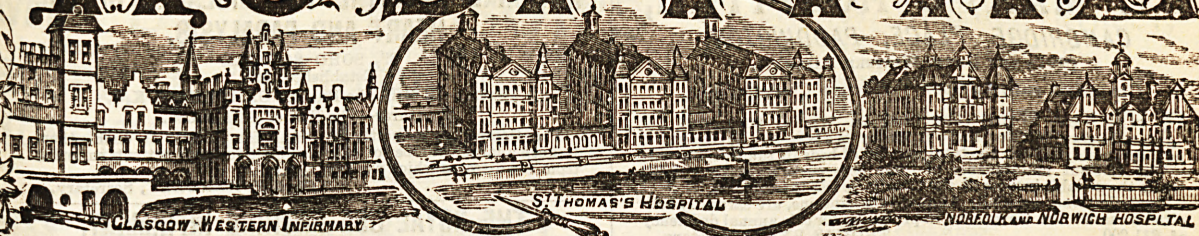

A WEEKLY JOURNAL OF

### Science, Medicine, Nursing and Philanthropy.

SATURDAY, JANUARY 10th, 1891.

**Doctors and the Peerage** - 227
**Passing Topics.**—New Loves and Old - 228
**The Doctor's Armchair.**—"Poisons as Required" - 229
**The Infirmary Medical Superintendents' Society** - 230
**The Microscope in Medicine.** By FRANK J. WETHERED, M.D.—I. Introductory - 231
**The History and Capabilities of Herbal Simples.** By W. T. FERNIE, M.D.—XXVI. Currants - 232
**Everybody's Page** - 233
**Notes and News** - 234
**Annotations.**—Mr. John Marshall, F.R.S.—Sir Richard Quain—Slaughtered Women and Children - 236
**Scraps and Gleanings** - 242
**The Practitioner's Mirror and Retrospect:—**
MEDICINE.—Loeffler on Diphtheria—Intratracheal Injections—Scarlatina without Fever—Hysterical Aphonia, 237
SURGERY. — Hypertrophy of the Prostate Gland, 238; Growth of Femur after Resection of Knee-Joint—Spina Ventosa—Spina Bifida, 239; Ganglions—Treatment of Club-foot, 240; Treatment of Flat-foot - 241
DIETETICS.—Mothers' Milk—Infants' Food - 241
NEW DRUGS, APPLIANCES, AND THINGS MEDICAL.—Antiseptic Requisites—Vichy Liqueur - 242
**The Student of Medicine.**—Students' Medical Societies—The Students' Bookshelf—Pass Lists—Athletics—Events for the Month of January.

### EXTRA SUPPLEMENT—THE NURSING MIRROR.

**En Passant.**—Biblewomen and Nurses—The Hatchet Buried—Mary Adelaide Letter—Short Items—The Nurses' Co-operation—Personal News—Lewis's Nursing Charts—A Nurse in Trouble—"Bluebeard" at Berry Wood - lxxvii

**Lectures on Surgical Ward Work and Nursing**—By ALEX. MILES, M.B.Edin., C.M., F.R.C.S.E.—Lecture IX.—The Dressing of Wounds - lxxviii

**Queen Victoria Jubilee Institute for Nurses** - lxxix

**Appointments** - lxxix

**Death in our Ranks** - lxxix

**For Reading to the Sick.**—The Old Year - lxxix

**Keeping Christmas** - lxxx

**Off Duty.**—The Brimsby Ghost (*concluded*) - lxxxii

**Where to Go.** - lxxxii

**Presentations—Notes and Queries** - lxxxii

**Amusements and Relaxation** - lxxxii

